# Co-Designed Online Training Program for Worry Management: The Role of Young People With Lived Experience of Worry in Program Development

**DOI:** 10.2196/66461

**Published:** 2025-05-21

**Authors:** Jessica Steward, Michelle L Moulds, Colette R Hirsch

**Affiliations:** 1 Institute of Psychiatry, Psychology and Neuroscience, King's College London, 16 De Crespigny Park, London, SE5 8AF, United Kingdom, 44 7947279322; 2 School of Psychology, UNSW Sydney, Sydney, New South Wales, Australia; 3 South London and Maudsley NHS Foundation Trust, London, United Kingdom

**Keywords:** digital intervention, mental health, patient and public involvement, young people, adolescent, experts by experience, participatory, anxiety, development, design, user experience

## Abstract

**Background:**

Many young people report high levels of worry, highlighting the need for interventions that teach strategies to help them shift focus away from worry. To maximize uptake by this population, interventions should be brief and accessible; to maximize dissemination, they should have potential for delivery at scale. We produced a multisession, online training program, Shift Focus, co-designed with young people with lived experience of worry. The online training program was accessed via a mobile app. In this paper, we describe how Lived Experience Advisory Panel (LEAP) members were involved in each stage of the process of developing the Shift Focus online training program, from refining session content through to designing and testing the online training program prototype.

**Objective:**

We aimed to engage with young people with lived experience of worry, to help refine, further develop, and tailor a new online training program designed to help shift focus away from worry.

**Methods:**

We recruited LEAP members (aged 16‐25 y) with lived experience of worry from diverse backgrounds across the United Kingdom. We used a highly iterative participatory design process, such that LEAP members provided input during all 4 phases of program development: refining and further developing session content, piloting sessions, developing user experience design, and testing the online training program prototype.

**Results:**

Feedback from LEAP members during each phase of the online platform development informed key decisions regarding the platform content, functionality, and the interface design to ensure it suited our target population. In phase 1, we learned that the platform needed to be simple and aesthetically pleasing, personalized to individual needs and preferences, accessible to all, track progress, and provide individuals with a sense of community with others with similar lived experiences. In phase 2, we learned that the platform also needed to provide further guidance on how to apply the Shift Focus techniques to daily life, using personalized reminder settings. In phase 3, we additionally learned that ease of navigation and interactivity were key to maintaining user engagement. The importance of program tracking was reiterated, as well as the need for accessibility settings to support all learning styles. In phase 4, we identified that technical problems with the online platform were a barrier to engagement. The inclusion of future iterations (eg, reward systems) to help promote engagement was suggested by LEAP members in multiple phases.

**Conclusions:**

LEAP members brought unique expertise and made key contributions to the development of the Shift Focus online training program and were highly valued members of the team. A highly iterative participatory design process enabled continuous feedback from LEAP members throughout, ensuring that their input was meaningful and that their key messages and ideas were incorporated into the final program.

## Introduction

Many young people experience high levels of uncontrollable worry [1], which can lead to great distress and be linked to psychological disorders such as generalized anxiety disorder (GAD). GAD is characterized by excessive and uncontrollable worries about everyday events or activities that are out of proportion to the inherent risk [2]. Uncontrollable worry takes up limited capacity resources, and such resources are needed for individuals to shift their focus from worry to other tasks or streams of thinking [3]. Given this, high worriers may benefit from cognitive techniques designed to shift attention away from worry.

Experimental studies provide preliminary support for this possibility. Eagleson et al [4] trained individuals presenting with GAD to shift focus away from worry by thinking about positive outcomes of their worries in either imagery or verbal based form, while participants in the control group were trained to shift focus away from worry by generating an unrelated positive image which was not associated with their worries. Participants practiced their designated technique for 1 week. In all 3 conditions, participants reported fewer negative intrusive thoughts and lower scores on self-reported measures of worry and anxiety at follow-up. Given this, although originally conceived as a control condition, generating an unrelated positive image appeared to be helpful for adults presenting with worry. Sawjani et al [5] extended this study and demonstrated that high worriers could be trained (in a single session) to generate an unrelated positive image to stop worry in the short term.

Given that worry is a difficulty experienced by many young people, we built on and extended the single session training developed by Sawjani et al [5] to develop a multisession online training program (Shift Focus), specifically tailored for young people with high levels of worry. Shift Focus is comprised of 8 brief sessions, which include psychoeducation about worry, guided imagery exercises, and within-session exercises practicing shifting focus away from worry and teaching ways to apply the skills in daily life.

We reasoned that an intervention for young people should be brief and accessible, and also have scope for flexible delivery. Digital health interventions (DHIs) offer such flexibility, providing the opportunity for this population to access evidence-based psychological interventions at a time and place of their choosing. Moreover, mobile apps promote independent skills practice in real time, enabling the application of techniques when they are most needed in day-to-day life. Accordingly, we developed an online training program that was accessed via a mobile app, as a means by which to teach young people cognitive strategies to shift their focus away from worry when they want to.

Despite the abovementioned benefits of DHI, recent reviews have found that while effective, they typically have poor uptake and service user engagement [6,7]—limiting their capacity to deliver longer-term mental health benefits. Engaging stakeholders in the development and tailoring of DHI is essential to maximize engagement and improve acceptability. Working closely with individuals with lived experience is a crucial element in developing new acceptable DHI. Accordingly, we recruited and engaged a panel of young people with lived experience of worry (a Lived Experience Advisory Panel [LEAP]) to obtain their feedback on Shift Focus, and leveraged their invaluable insights to refine and further develop the program.

Patient and public involvement (PPI) in research has become increasingly common internationally [8], and refers to contributions from people with lived experience of a psychological or physical health condition under study, including input into how research is designed, conducted, and disseminated [9]. Different forms of knowledge and expertise can then be brought together, eradicating traditional divides between researchers and service users that in the past may have been grounded in assumptions which differentiate “expert” researchers from people with lived experience, traditionally considered nonexpert. There has been some criticism of PPI initiatives because they are too narrow and tokenistic in nature (eg, [10,11]), and in turn, a call for PPI members to be more empowered within research settings [12]. Encouragingly, there is evidence that meaningful PPI engagement improves the quality of research, ensuring that it is relevant to the needs of the community, thus enabling more positive impact [13].

Our goal in this paper is to outline the range of ways in which our LEAP members contributed to the process of developing, co-designing, and testing the acceptability of Shift Focus, an online, standalone, multisession training program (delivered via a mobile app) for young people (16‐25 y) who report difficulty stopping worry. Specifically, we wanted to determine whether the online training program was acceptable to our target population of young people. To do so, we sought feedback on LEAP members’ satisfaction with the program and willingness to engage, and on their perceptions of the program’s capacity to help them manage worry. We obtained feedback on the content, look, and feel of the online training program over different phases of development, with the goals of maximizing potential user satisfaction and engagement of the target population when they are offered the finalized version of the online training.

LEAP members’ involvement was made up of 4 phases. The aim of phase 1 was to develop session content. Specifically, LEAP members provided feedback on the sessions produced by the researchers to further develop and refine session content. Additionally, we sought feedback on the acceptability of the intervention and sought feedback to help identify and address any barriers to young people’s understanding of session content. Informed by this feedback, we modified the intervention with the goal of building sessions that were tailored to young people’s needs and preferences.

In phase 2 we explored feasibility; that is, the online training program’s practicality and the ease with which young people could implement it in their daily lives. Specifically, we were interested in whether young people had the time and resources to engage in the program, as well as their views on whether it was feasible that they would use the techniques taught in the program in day-to-day life. We also obtained LEAP feedback on the modified content (ie, changes made in response to feedback in phase 1) and additions (eg, push notifications), and explored acceptability.

The aim of phase 3 was to develop user experience (UX) design. This involved engaging in a user journey mapping workshop with UX designers to identify what users see, think, feel, and do when they interact with the online platform, and to obtain feedback on any difficulties that they experienced. The purpose of the workshop was to ensure that the UX designers had a clear understanding of the user group. Phase 3, therefore, involved an iterative process with LEAP members and UX designers, so that the online platform would be suited to young people’s needs and preferences.

The final phase (phase 4) involved an online training program testing. LEAP members provided final feedback on the online platform. Our goal was to identify and resolve any final technical issues and obtain further feedback on LEAP members’ experiences in reviewing the sessions.

## Methods

### Overview

**Figure 1.**
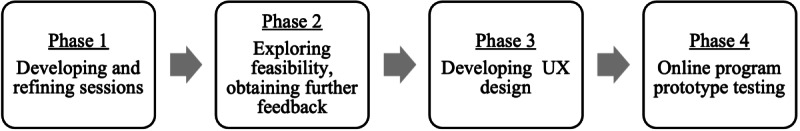
Phases of development of the multisession online training program. UX: user experience.

LEAP consultation and online training program development took place from September 2022 to December 2023 (Figure 1). There were 4 phases of LEAP involvement: developing and refining session content (phase 1), exploring the feasibility of completing the training and obtaining feedback on modified content (phase 2), developing UX design (phase 3), and testing the online program prototype (phase 4). The highly iterative participatory design process enabled continuous feedback from LEAP members during all phases of program development.

LEAP members were consulted online. New LEAP members joined at the beginning of phases 1, 2, and 4. This was to ensure we had diverse perspectives and insights from different groups of young people, enriching the development process. Phase 3 LEAP members were a subset of those from phase 1. We invited this subgroup to provide feedback on the UX design of the online platform in phase 3 on the basis that their strong familiarity with the session content would enrich their feedback during the UX design phase.

### Ethical Considerations

The research project was approved by King’s College Research Ethics Committee (HR-19/20‐14855). LEAP members were consulted to co-design the Shift Focus online training program, and as such, were not research participants per se; therefore, they did not need to provide informed consent. Nonetheless, LEAP members fully understood their role in the development of the project and the reason that they were being consulted and asked to provide feedback; hence, they were fully informed regarding their involvement. The information LEAP members provided is stored in an anonymized form on King’s College London servers. LEAP members were reimbursed £20 (US$ 22.55) per hour for their time.

## Phase 1: Developing Sessions

### Methods

Ten LEAP members (aged 16‐25 y; female n=8, male n=2) who self-identified as worriers were recruited from Child and Adolescent Mental Health Services (CAMHS) in South London and Maudsley NHS Foundation Trust (SLaM) and a Digital Research Advisory Group in Greater Manchester Mental Health NHS Foundation Trust. JS and CRH presented the LEAP opportunity at the Digital Research Advisory Group meeting and asked young people to make contact via email if they were interested in contributing to the project. The opportunity to be part of the LEAP was featured in the SLaM monthly newsletter, which is distributed to SLaM clinicians, who then shared the poster with appropriate service users in local CAMHS services. With consent, the clinicians provided the service users’ email addresses to JS, who made contact with potential LEAP members to explain what the role would involve. We also recruited from the general community via posters (eg, placed on local bulletin boards in South London) and social media paid advertisements (eg, posted on Facebook [Meta] and Instagram [Meta]).

Most LEAP members were currently accessing or had previously accessed CAMHS. LEAP members were from a diverse range of ethnic backgrounds (groupings in this paper were based on free text descriptions of their ethnic identity, which were then mapped to Office for National Statistics Census Categories [14][Asian or British Asian background: n=3; Black African background n=1; White background: n=6]). LEAP members completed the Penn State Worry Questionnaire (PSWQ) [15] to indicate their level of worry.

Shift Focus is comprised of 8 brief sessions developed by the research team and programmed on the online survey platform, Qualtrics. JS met with LEAP members individually via Microsoft Teams (Microsoft Corp) and provided a brief overview of the intervention. LEAP members accessed the intervention sessions through a Qualtrics link, shared their screens with JS, and provided feedback on the sessions. LEAP members were given the option of either sharing their thoughts about the sessions as they worked through them or providing feedback at the end of the session. JS recorded the time it took each LEAP member to complete a given session (mean=10 min and 4 s; SD=13.7 s). LEAP members answered questions on the acceptability and feasibility of completing each session (Multimedia Appendix 1). Low scores on the scales indicated poor acceptability or feasibility of use, which was then followed up with detailed discussion.

In parallel with phase 1 and phase 2, we collaborated with a trainee clinical psychologist at Oxford University to create animations to explain key psychological concepts within sessions. We prioritized psychological concepts that were difficult to understand, as well as concepts that LEAP members particularly recommended that we represent in animation form. LEAP members provided ideas for the visuals and feedback on drafts of the animations before they were finalized.

### Results

#### Overview

Table 1 presents examples of some of the feedback obtained from LEAP members in phase 1. We note whether feedback was implemented; when it was not implemented, we provide our rationale. This feedback led to major session revisions and requests for additional LEAP input. Feasible changes were implemented before phase 2.

Key learnings from phase 1 LEAP consultations were that the online training program should: (1) be simple and aesthetically pleasing, (2) be personalized, (3) support all learning styles, (4) track progress, (5) provide a sense of community, (6) provide guidance on postsession activities, and (7) have a clear rationale, aims, and objectives. LEAP members also suggested changes to the sessions to make them more accessible.

**Table 1.** Examples of feedback provided by LEAP^a^ members during phase 1.

LEAP members	Feedback	Implementation
LEAP member 1	“Recap would be great because even if the sessions are not too long it still feels like a lot of information to take in. This would ensure I don’t feel overwhelmed with the new information.”	Yes (January 30, 2023). Recaps were implemented throughout sessions.
LEAP member 1	“It would be good to have more categories added to the worry themes. Sometimes people have very specific worries, so would be good to have fewer generic worries. Other themes could include anxieties about leaving the house, worries about the future or how successful their career is (there is lots of pressure on young people to achieve highly).”	Yes (March 3, 2023). Feedback added to session content and noted on UX^b^ design document for phase 3.
LEAP member 2	“Maybe try to be more specific about identifying certain situations where we are worrying less. For example, if I’m at school, I could be prompted to think about times when I am worrying less. If it is more general in the session content, I might not think about it.”	No (March 8, 2023). JS discussed this with the research team. While we understood the motivation for the suggestion, we had concerns about priming an episode of worry. For example, if we remind young people when they are not worrying, they might start to worry. Therefore, we opted to leave it up to young people to notice times when they are not worrying in their daily life, and allowed them to add activities to the online platform if they chose.

a LEAP: Lived Experience Advisory Panel.

b UX: user experience.

#### Simple and Aesthetically Pleasing

LEAP members strongly emphasized that the online platform interface should be simple to navigate and that text should be easy to comprehend. It was clear that an online platform that was not easy to use during skills practice would not be used. They also requested an aesthetically pleasing design with distinct colors for different sections of the platform. Accordingly, we condensed the text, simplified psychological constructs, and noted their ideas for the UX design in phase 3.

#### Personalized to Individual Needs and Preferences

LEAP members indicated that the online platform should be tailored to individual needs and feel personalized. We added features for users to set a personal username on the homepage, reflect on their own worries, and upload personal images for exercises in sessions during phase 3. Budget constraints prevented us from implementing personal profiles and mood tracking features. LEAP members also requested gender-specific voice options for audio exercises, reasoning that some participants may feel more at ease with a specific gender. We noted these items for future directions in a larger study.

LEAP members also requested personal reminder settings (ie, being able to choose when the reminders from the online platform arrived). LEAP members reported that this would increase engagement because reminders would not arrive at inconvenient times, and it would allow them to feel autonomous over their skill development. We implemented this idea during phase 3.

#### Support All Learning Styles

LEAP members emphasized the importance of the session content being accessible to all, particularly neurodiverse individuals. They suggested that this could be achieved by including multimodal formats (written text, videos, audios, and visuals) to support all learning styles. We decided to create animations to explain key psychological concepts, add bold text for visual learners, and use an audio format instead of text format when possible. We also added optional recap animations at the start of sessions to help consolidate key psychoeducation concepts. LEAP members also requested an audio book feature and the ability to review previous session content, however, this could not be implemented due to budget constraints.

The repetitive nature of exercises within sessions was identified as a significant barrier to engagement. To address this, we minimized identical content and created an animation to explain why repetition of session exercises was important. We also improved session pacing to help users absorb the material, reduce the repetitiveness of exercises, and avoid overwhelming with session content. We introduced a 48-hour break between sessions 4, 5, 6, and 7. Users were asked to practice the skills from sessions during these intervals to embed the skills they had learned.

#### Tracks Progress

Tracking progress was identified as essential for engagement. We created a session tracking page with a clear timeline, added progress bars, and end-of-session summaries that outlined how the future session would build upon the previous session. Several progress tracking features were out of scope. Most LEAP members requested a reward system within a personal profile on the online platform (eg, collecting points or rewards if they engaged with the mobile app regularly). This feature was noted as essential for a larger study.

#### Provides a Sense of Community and Connection to Others

LEAP members emphasized a need for a sense of community on the online platform and requested a space to engage with other young people who experience anxiety. Due to limited capacity for moderation, the addition of a community section was not feasible in this study. With permission, we added examples based on LEAP members’ experiences within sessions to validate their experience.

LEAP members requested personal audio exercises for each session, referencing other platforms on which familiar figures provided friendly introductions at the beginning of sessions to create a welcoming atmosphere. They also noted that this could reduce the repetitiveness of exercises across sessions. Based on this feedback, JS provided tailored introductions for each session’s audio exercises.

LEAP members also sought validation for their progress and acknowledgment that difficulty working through the content and exercises was normal. To address this, we provided validation and normalized challenges through written text and audio files in the sessions.

#### Guidance on Postsession Activities

LEAP members requested guidance on postsession activities, raising concerns that they may forget to resume their daily tasks after a session by staying on the online platform or continuing to worry. In response, we created an interactive activity panel at the end of sessions during phase 3, which encouraged users to choose an engaging activity to complete after the session to help them return to daily life and step away from their electronic devices.

#### Clear Rationale, Aims, and Objectives

LEAP members requested a more detailed rationale for the session content, including clear aims and objectives. In response, we added additional psychoeducation, a detailed rationale for the skills taught, and more information about research on the topic of managing worry.

## Phase 2: Exploring Feasibility of Completing the Training and Obtaining Feedback on Modified Content

### Methods

Ten new LEAP members aged 16‐25 years (female n=16 and male n=4) were recruited through the King’s College London Research Circular Advertisement. LEAP members self-selected by completing a screening questionnaire (eligible if they scored ≤28 on the PSWQ) and completed a study interest call with a member of the research team. LEAP members were from a diverse range of ethnic backgrounds (Asian or Asian British background n=6, Black African background n=1, White background n=10, mixed or multiple ethic background n=1, other ethnic background n=1, and unknown n=1).

LEAP members were given access to the Shift Focus sessions and asked to complete them within 4 weeks. The intervention was comprised of eight 10-minute sessions delivered via Qualtrics, which participants accessed via their mobile phones. JS manually granted session access and sent text message reminders to simulate future platform notifications. LEAP members also participated in online exit interviews to provide feedback on the sessions.

### Results

#### Overview

The key messages from LEAP consultations in phase 2 was that the program should (1) provide guidance on how to apply the skills to daily life, and further, that we should: (2) reframe homework tasks, (3) adapt the personal reminder settings and (4) make further tweaks to the Shift Focus techniques. Most feedback was implemented, except in cases where research budget constraints meant that the suggested changes were not feasible.

#### Guidance on How to Apply the Skills to Daily Life

LEAP members reported needing additional support on how to apply session skills in daily life, especially in nonquiet spaces such as public transport. In response, we created a ninth and final session to provide guidance on how to apply the skills in everyday situations.

#### Reframe Homework Tasks

Most LEAP members understood the purpose of homework exercises between sessions. Some LEAP members completed the homework exercises while others did not. A few LEAP members reported that they found it difficult to find the motivation to engage with the homework exercises. Our goal was to prevent users from viewing homework as a chore. We carefully chose our language and introduced new terminology to replace the word “homework,” and designed a new part of the online training platform called the Shift Focus Training Hub. Users were asked to visit the Hub to complete 2 different between-session exercises. LEAP members also reported that they found it less motivating to complete homework exercises because they could not track their homework completion. It was clear that integrating a reward system and progress tracker into the Shift Focus Training Hub would be essential to increase engagement in a future study.

#### Adapt Personal Reminder Settings

We discussed the notion of “alarm fatigue” through over-notification with software developers because this was identified as a barrier to engagement in phase 2. In response, users could choose reminder frequency (1‐3 daily) and timing (8 AM, 3 PM, and 8 PM) for homework exercises. This idea was implemented during phase 3.

#### Modifications to the Shift Focus Techniques

We made several changes to the Shift Focus techniques following LEAP feedback in phase 2. One technique involved asking LEAP members to identify activities that they felt were particularly absorbing, and thus less impacted by worry. They appreciated reflecting on this and requested more integration of this concept into the sessions. We also iteratively adapted the final session content and significantly modified the audio exercises based on feedback. LEAP members also helped identify aspects of the techniques that could temporarily increase worry, leading to content adjustments to minimize this risk.

In addition to these new recommendations, LEAP members reiterated ideas expressed by phase 1 LEAP members, namely: the online platform must be personalized to individual needs and preferences (including personal reminder settings) and include progress tracking features. This reinforced the importance of implementing these ideas during phase 3, within the constraints of our research budget.

## Phase 3: Developing UX Design

### Methods

Six LEAP members aged 16‐25 years (female n=4, male n=2) from phase 1 (see above for recruitment approach) provided further feedback in phase 3. LEAP members were from an Asian or Asian British background (n=3) and a White background (n=3).

**Figure 2.**
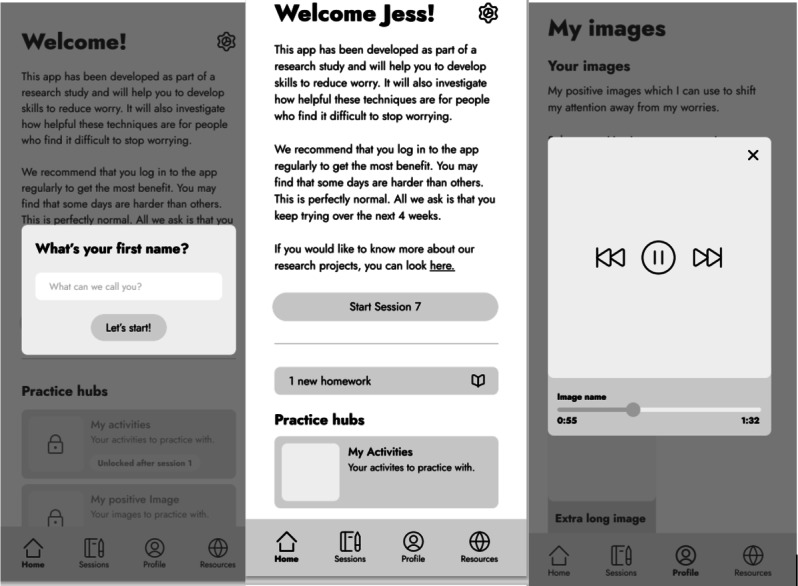
Examples of wireframes from the online training program.

The UX design team created wireframes (Figure 2) following the user journey mapping workshop with CRH and JS. Wireframing is a process of creating black-and-white images of the online platform on screens and arranging them to determine how users would navigate the platform. It focuses on the functionality and layout of the online platform.

JS presented the wireframes to LEAP members individually via Microsoft Teams. JS explained a scenario in which the online platform was recommended to them by a friend, and they were using it for the first time. Using the think-aloud approach [16], LEAP members shared their screens, navigated through the wireframes, and voiced their thoughts. JS noted their feedback and identified navigation issues. The responses were then collated, and feasible feedback was incorporated by the UX designers before moving onto the user interface (UI) design phase.

The designers presented the initial UI designs (look and feel) of the online platform, including functionality, layout, and color schemes. JS repeated the process with LEAP members, gathering feedback on the UI design. The designers incorporated feasible feedback and then presented final designs to the research team for the last amendments.

### Results

#### Overview

Key messages from LEAP consultations in phase 3 were that the online platform should be (1) simple to navigate, (2) interactive and engaging, and (3) track progress. LEAP members requested that the platform should have: (4) accessibility settings and (5) provide a sense of community. LEAP members additionally (6) provided extensive feedback on the look and feel of the online platform and (7) noted key changes to session content.

#### Simple to Navigate

A think-aloud approach was crucial to determine how LEAP members found navigating the online platform. LEAP members reported areas that they found difficult to navigate or confusing. It was clear that an online platform that was simple to use and navigate was essential for engagement.

#### Interactive and Engaging

LEAP members requested more interactive and engaging features on the platform. For example, LEAP members liked the reminder buttons, which were designed with different colors and icons for morning (yellow with a sun icon) and evening (dark blue with a moon icon) to enhance user engagement with personal reminder settings. LEAP members also liked being able to upload personal images for tasks that could be seen as interactive tiles which they could flip around to add their own descriptions of what it meant to them.

#### Track Progress

LEAP members requested a progress tracking bar within sessions to motivate users to finish their sessions. LEAP members also wanted to track how many times they had completed exercises on the online platform. We added a progress bar and session timeline page, but could not implement exercise tracking due to budget constraints. Progress tracking and a reward system were noted as essential for a larger study.

#### Accessibility Settings

LEAP members reinforced the need for the online platform to be accessible to all and suggested including features such as adjustable text size and dark or light modes so that participants could reduce the brightness if needed. Although these suggestions were noted for a larger study, they could not be implemented due to budget constraints. In phase 1, LEAP members requested transcripts for audio clips to improve accessibility. However, in phase 3, LEAP members found this particular multimodal format unhelpful and requested that the transcripts be removed, which we did.

#### Sense of Community

LEAP members requested a space on the platform to engage with other young people who experience worry. As noted previously, we did not have the capacity for a moderator, so this could not be implemented. As an alternative, we proposed a community section that reported the number of young people who had completed the Shift Focus program on a given day. LEAP members reported that this could create a sense of competition, resulting in them worrying about how quickly they should be completing the program. Consequently, we decided not to implement tracking of program completion in a community section.

#### Look and Feel

**Figure 3.**
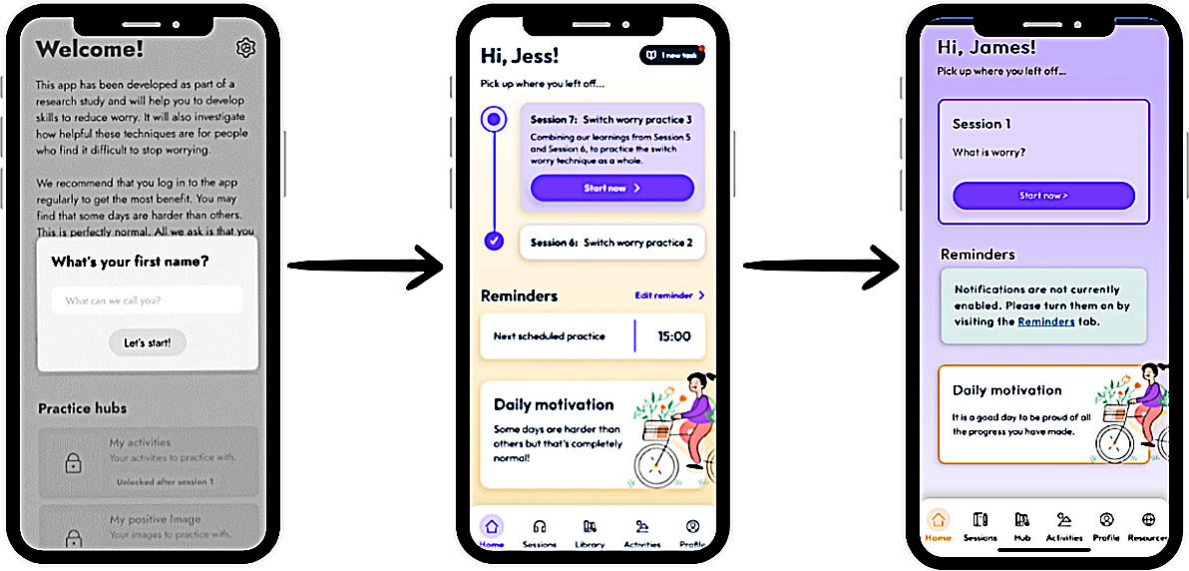
Images comparing a wireframe, preliminary design, and final design of the homepage that incorporated LEAP feedback. LEAP: Lived Experience Advisory Panel.

LEAP members provided extensive feedback on the color schemes for the online platform. LEAP members did not find our first color scheme for the platform aesthetically pleasing. For example, they found the color white too bright, and they did not like the orange-colored reminder boxes because it felt was like an amber warning (ie, made them feel as though they had done something wrong). LEAP members also requested clearer headers and page structure. In response, we revised the colors and page layout (Figures3  4). Budget constraints prevented us from allowing users to choose their own background color; this feature was noted for inclusion in a larger future study.

**Figure 4.**
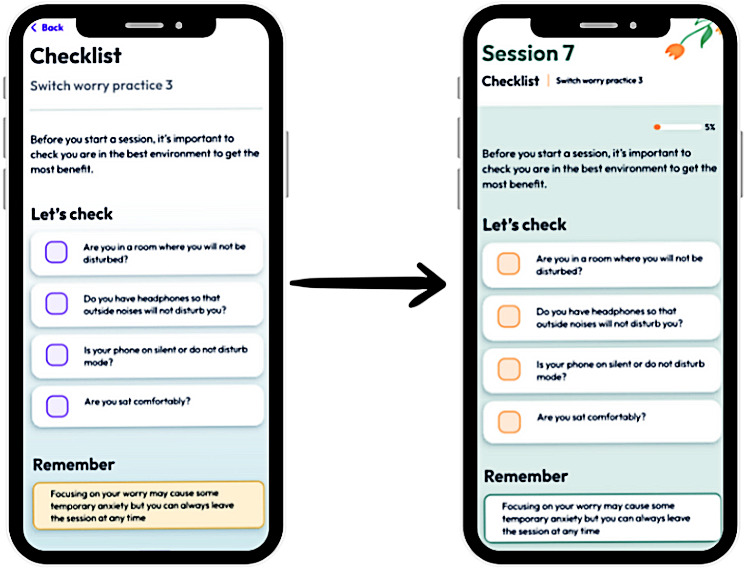
Images comparing an original session design and a final design that incorporated LEAP feedback. LEAP: Lived Experience Advisory Panel.

#### Key Changes to Session Content

LEAP members highlighted session content they did not like or found confusing. For example, LEAP members reported that overuse of the phrase “well done!” felt patronizing. We revised the session content in response to this feedback.

## Phase 4: Online Training Program Testing

### Methods

Eight new LEAP members aged 16‐25 years (female n=6, male n=2) were recruited through the Shift Focus social media page on Instagram to provide feedback on the online training program prototype. All LEAP members self-selected as being worriers from the social media advertisement. The group was predominantly White (n=7) with 1 member from another ethnic background (n=1). In phase 4, we used the Repetitive Thinking Questionnaire to measure repetitive negative thinking, as the PSWQ is for those aged 18 years and older and so does not cover the full age range for those who will be ultimately offered the online training program. The Repetitive Thinking Questionnaire is validated in both adult and adolescent samples [17,18].

LEAP members accessed the Shift Focus sessions over 4 weeks. The intervention was comprised of nine 10-minute sessions delivered via the online platform. LEAP members had the option of setting personal reminders for homework exercises and received push notifications for new sessions or inactivity. LEAP members were interviewed after completing the four weeks of the online training program to identify any technical problems or engagement issues before study launch. Feedback that could not be implemented due to time constraints was noted for future studies.

### Results

#### Overview

The key messages from LEAP consultations in phase 4 were: (1) technical problems hindered engagement, (2) users may have data security concerns, and (3) the need for a platform tutorial. LEAP members also suggested (4) further changes to session content in a future study.

#### Technical Problems

LEAP consultations in phase 4 identified technical problems with the online platform, including malfunctioning buttons and missed reminders. Technical glitches were a barrier to engagement as LEAP members who experienced technical problems needed more reminders from the research team to complete sessions. Piloting the prototype to identify and fix technical issues before study launch was essential for this reason.

#### Data Security Concerns

A LEAP member reported that it felt strange uploading their own personal images to the online platform and highlighted the importance of data security concerns among users. The research team subsequently addressed this by providing clear information about data security and storage in the study information sheet. In any future study, we will explicitly highlight these details on the online platform to alleviate such concerns.

#### Platform Tutorial

LEAP members found it confusing that they had access to all sections of the online platform from the outset, particularly given that some areas of the platform were not needed until later sessions. LEAP members suggested there was a video tutorial on platform navigation at the start, rather than introducing areas of the platform as they worked through sessions. This will be implemented in a future study.

#### Session Content Changes

LEAP members also proposed further changes we could make to the session content in a future study. This included adding more guidance on how to apply the skills to daily life, a clearer emphasis on the long-term benefits of using the skills, more varied sessions with less repetition, and additional resources for independent learning (eg, signposting articles). This was invaluable feedback that will be implemented in a future study.

## Discussion

### Principal Findings

The Shift Focus online platform was developed, designed, and tested with psychologists, software developers, and young people with lived experience of worry. We aimed to engage with young people with lived experience of worry, to refine and further develop a new online training program designed to help young people shift focus away from worry. We adopted a highly iterative participatory design process across 4 phases—an approach that ensured LEAP members were integral to all stages of the Shift Focus platform development, and that the online platform was tailored and personalized to young people’s needs and preferences.

In phase 1, we learned that the online platform needed to be simple to use, interactive, aesthetically pleasing, and personalized to individual needs and preferences. This echoes existing research which emphasizes the importance of aesthetics [19], simplicity [20], interactivity [21], and personalization [22] when designing DHI’s. It was also important that the platform was accessible, tracked progress, and provided a sense of community with others with shared lived experience of worry.

In phase 2, we learned that the platform needed to provide further guidance on how to apply the Shift Focus techniques to daily life and have personalized reminder settings. LEAP members also suggested (in multiple phases) that we should include enhanced progress tracking features and a reward system in a future iteration of the Shift Focus online training program to promote engagement. This is consistent with findings that suggest that gamification, such as points or incentives, can motivate consistent use and adherence to DHIs [23] .

In phase 3, LEAP members provided invaluable feedback on the appearance and functionality of the online platform, which played a crucial role in shaping the “look and feel” of the online training platform. Similarly to phase 1, LEAP members re-emphasized the importance of a community section on the platform to connect with other young people with shared lived experience of worry. Clearly, a sense of belonging and social connection is a crucial consideration when developing DHIs for young people.

In phase 4, we identified that technical problems with the online platform were barriers to engagement. Technical issues have been widely reported as a barrier to engagement in DHI research [24] . Additionally, data security concerns with the online training platform were raised by a LEAP member. This highlights the importance of clearly outlining how data is stored to DHI users to establish user trust and engagement.

We put the voice of lived experience at the center of the project by ensuring key messages from LEAP were incorporated from phases 1-4. LEAP members were very effective at helping the research team implement gentle shifts in language and refine explanations of psychological concepts to make them more accessible to our target population. Our LEAP members ranged in age from 16 to 25 years, and we observed significant differences in the preferences of the younger (16-18 years) and older (23-25 years) members. For example, the younger members preferred simplified text with bullet points, while the older members expressed a preference for more reflective questions and detailed session rationales. This large age gap was challenging at times due to the different preferences that were sometimes diametrically opposed. In the future, we will explore the possibility of developing age-specific versions of the online platform to address these varied needs and preferences.

Power differentials and research hierarchies have been reported as barriers to working collaboratively and developing meaningful working relationships with LEAP members [25,26]. We found that encouraging LEAP members to be open and critical of our research was important to gain the most from their insights and reduce power dynamics. We regularly emphasized to LEAP members that they brought unique expertise to the team and how valuable it was for them to challenge our research ideas and ways of thinking. We also explained how any negative feedback provided was very helpful because it highlighted our blind spots and areas in which we could improve. Our goal was to create a space for LEAP members to be critical of our work and session content, to enable them to feel that their expertise was valued.

LEAP initiatives have faced criticism for being too narrow and tokenistic [10,11], leading to calls for greater empowerment of LEAP members within research settings [12]. Therefore, it is essential for research teams to implement research processes that reduce the possibility of tokenism. We created a table to log our rationale for not implementing ideas suggested by LEAP members in phase 1. This kept the research team accountable and ensured that we did not just implement suggestions from LEAP consultations based on our own personal preferences.

Additionally, it was important that we were transparent with LEAP members when we could not implement an idea due to financial constraints within our research project. Managing expectations was key, and being clear about what was out of scope was important to ensure that LEAP members did not think their ideas were ignored. If we were unable to implement an idea, we worked collaboratively with the LEAP member to consider possible ways we could address the issue raised, even in part, within the scope of our budget. LEAP members valued our honesty and had a stronger understanding of the research team’s decision-making processes, which we believe increased engagement.

It was important to ask LEAP members whether they felt like valued members of the research team to ensure that we learned from the process and could change our practices to be more inclusive if necessary. Focusing only on the impact LEAP has on research, without considering the potential benefits or costs the work has on the LEAP members involved, overlooks an essential part of the process [27,28]. Therefore, we sought feedback on this via informal discussions and anonymous feedback questionnaires.

During phase 3, the UX designers and the LEAP members generated many excellent ideas for the design of the online platform. This sometimes resulted in competing ideas, which made decision-making during the design phase challenging. We found it helpful to clarify with LEAP members why they favored certain UX design features over others, rather than simply being informed of their likes and dislikes. This ensured the design of the online platform was flexible for UX designers, but importantly, also met the needs of young people.

This study has limitations. We invited any young people aged 16‐25 years to participate if they classified themselves as being a high worrier. With the exception of phase 2, we did not use a cutoff on a self-report measure of worry to determine eligibility. Furthermore, in phases 1 and 3 (same individuals in both phases), six LEAP members had moderate but not high levels of worry, and in phase 4, one LEAP member did not have high levels of repetitive thinking on the standardized questionnaire. As a result, it is possible that feedback from these individuals did not reflect the views of high worriers who are the target population. That said, they were aware that they were providing feedback for an online training program designed to help high worriers. Additionally, JS created a table to document all the feedback from LEAP members in phase 1 and whether the feedback was implemented. This process was not used in phases 2-4. Future studies should plan to generate feedback implementation tables consistently across all phases. Furthermore, we did not present the phase 1 feedback implementation table to the LEAP members, and we suggest that this be done in future work to enhance their understanding of our decision-making processes.

### Conclusion

LEAP members brought unique expertise grounded in their lived experience of worry. LEAP members were highly valued members of the team and were encouraged to give critical feedback at all stages. A highly iterative participatory co-design process enabled continuous feedback from LEAP members throughout, ensuring their input was meaningful and key messages and ideas were incorporated into the online platform. Study exit interviews from the experiment that will use the Shift Focus online program delivered via a mobile app will inform us as to whether the process led to us developing an engaging online training platform that worked well for young people (JS et al, unpublished data, 2025). Our Shift Focus study is one example of how having meaningful LEAP involvement in the development of DHIs benefits the research and intervention greatly.

## Supplementary material

10.2196/66461 Multimedia Appendix 1 Additional materials—questions asked in phase 1 regarding the content of each session.

## Abbreviations

CAMHS: Child and Adolescent Mental Health Services
DHI: digital health intervention
GAD: generalized anxiety disorder
LEAP: Lived Experience Advisory Panel
NHS: National Health Service
PPI: patient and public involvement
PSWQ: Penn State Worry Questionnaire
SLaM: South London and Maudsley NHS Foundation Trust
UI: user interface
UX: user experience
