# Supplementary material for: Co-Designed Online Training Program for Worry Management: The Role of Young People With Lived Experience of Worry in Program Development
Source: JMIR Form Res. 2025 May 21;9:e66461. doi: 10.2196/66461 (PMC12118939; doi:10.2196/66461)
Supplement: Multimedia Appendix 1 [file formative-v9-e66461-s001.pdf]

LEAP feedback Phase 1 – Shift Focus Training Content

SESSION 1: What is Worry?

Name:

Please rate each of the following items in relation to the app content you have just listened to with the research team. Each item is rated from 0 (strongly disagree) to 100 (strongly agree).

1. **The information I was given was understandable**

Strongly Disagree — Strongly Agree

0 10 20 30 40 50 60 70 80 90 100

How could we improve?

2. **I feel that most 16–25-year-olds experiencing worry and/or anxiety would be able to work through this content**

Strongly Disagree — Strongly Agree

0 10 20 30 40 50 60 70 80 90 100

How could we improve?

3. **There were concepts explained to me that I did not understand**

Strongly Disagree — Strongly Agree

0 10 20 30 40 50 60 70 80 90 100

Can you remember which concepts you did not understand?

4. **How likely would you be to try to notice situations where you weren't worrying?**

Not at all likely — Very likely

0 10 20 30 40 50 60 70 80 90 100

Please tell us more!

5. **How likely would you be to come back to complete the next session?**

Not at all likely **Very likely**

0	10	20	30	40	50	60	70	80	90	100

Why is this the case?

6. **Were there any parts of the session which you did not like?**

What section was this?

7. **Were there any parts of the content which you would have preferred to be presented as animations or video?**

Can you describe the content?

8. **What were three things you learnt from this session?**

1.

2.

3.

9. Is there any other feedback you would like to give?

Please tell us more!

SESSION 2: Let's get started!

Name:

Please rate each of the following items in relation to the app content you have just listened to with the research team. Each item is rated from 0 (strongly disagree) to 100 (strongly agree).

1. **The information I was given was understandable**

Strongly Disagree **Strongly Agree**

0 10 20 30 40 50 60 70 80 90 100

How could we improve?

2. **I feel that most 16–25-year-olds experiencing worry and/or anxiety would be able to work through this content**

Strongly Disagree **Strongly Agree**

0 10 20 30 40 50 60 70 80 90 100

How could we improve?

3. **There were concepts explained to me that I did not understand**

Strongly Disagree **Strongly Agree**

0 10 20 30 40 50 60 70 80 90 100

Can you remember which concepts you did not understand?

4. **Was there anything that you did which helped you generate your image or make your image clearer?**

Please tell us more!

5. How likely would you be to come back to complete the next session?

Not at all likely										Very likely
0	10	20	30	40	50	60	70	80	90	100

Why is this the case?

6. Were there any parts of the session which you did not like?

What section was this?

7. Were there any parts of the content which you would have preferred to be presented as animations or video?

Can you describe the content?

8. What were three things you learnt from this session?

1.<br><br>2.<br><br>3.

9. Is there any other feedback you would like to give?

Please tell us more!

SESSION 3: Let's get started!

Please rate each of the following items in relation to the app content you have just listened to with the research team. Each item is rated from 0 (strongly disagree) to 100 (strongly agree).

1. **The information I was given was understandable**

Strongly Disagree **Strongly Agree**

0 10 20 30 40 50 60 70 80 90 100

How could we improve?

2. **I feel that most 16–25-year-olds experiencing worry and/or anxiety would be able to work through this content**

Strongly Disagree **Strongly Agree**

0 10 20 30 40 50 60 70 80 90 100

How could we improve?

3. **There were concepts explained to me that I did not understand**

Strongly Disagree **Strongly Agree**

0 10 20 30 40 50 60 70 80 90 100

Can you remember which concepts you did not understand?

4. **Was there anything that you did which helped you create your image or make your image clearer?**

Please tell us more!

5. How did you find generating your own image with the audio?

Please tell us more!

6. How likely would you be to come back to complete the next session?

Not at all likely **Very likely**

0	10	20	30	40	50	60	70	80	90	100

Why is this the case?

7. Were there any parts of the session which you did not like?

What section was this?

8. Were there any parts of the content which you would have preferred to be presented as animations or video?

Can you describe the content?

9. What were three things you learnt from this session?

10. Is there any other feedback you would like to give?

Please tell us more!

SESSION 4

Name:

Please rate each of the following items in relation to the app content you have just listened to with the research team. Each item is rated from 0 (strongly disagree) to 100 (strongly agree).

1. **The information I was given was understandable**

Strongly Disagree ... Strongly Agree

0 10 20 30 40 50 60 70 80 90 100

How could we improve?

2. **I feel that most 16–25-year-olds experiencing worry and/or anxiety would be able to work through this content**

Strongly Disagree ... Strongly Agree

0 10 20 30 40 50 60 70 80 90 100

How could we improve?

3. **There were concepts explained to me that I did not understand**

Strongly Disagree ... Strongly Agree

0 10 20 30 40 50 60 70 80 90 100

Can you remember which concepts you did not understand?

4. **Was there anything that you did which helped you create your image or make your image clearer?**

Please tell us more!

5. **How did you find generating your own image with the audio?**

Please tell us more!

6. **How likely would you be to come back to complete the next session?**

Not at all likely **Very likely**

0 10 20 30 40 50 60 70 80 90 100

Why is this the case?

7. **Were there any parts of the session which you did not like?**

What section was this?

8. **Were there any parts of the content which you would have preferred to be presented as animations or video?**

Can you describe the content?

9. **What were three things you learnt from this session?**

10. **Is there any other feedback you would like to give?**

Please tell us more!

SESSION 5

Name:

Please rate each of the following items in relation to the app content you have just listened to with the research team. Each item is rated from 0 (strongly disagree) to 100 (strongly agree).

1. **The information I was given was understandable**

Strongly Disagree **Strongly Agree**

0 10 20 30 40 50 60 70 80 90 100

How could we improve?

2. **I feel that most 16–25-year-olds experiencing worry and/or anxiety would be able to work through this content**

Strongly Disagree **Strongly Agree**

0 10 20 30 40 50 60 70 80 90 100

How could we improve?

3. **There were concepts explained to me that I did not understand**

Strongly Disagree **Strongly Agree**

0 10 20 30 40 50 60 70 80 90 100

Can you remember which concepts you did not understand?

4. **Was there anything that you did which helped you create your image or make your image clearer?**

Please tell us more!

5. **How did you find the stop worry practice task?**

Please tell us more!

6. **How likely would you be to come back to complete the next session?**

Not at all likely **Very likely**

0 10 20 30 40 50 60 70 80 90 100

Why is this the case?

7. **Were there any parts of the session which you did not like?**

What section was this?

8. **Were there any parts of the content which you would have preferred to be presented as animations or video?**

Can you describe the content?

9. What were three things you learnt from this session?

10. Is there any other feedback you would like to give?

Please tell us more!

SESSION 6

Name:

Please rate each of the following items in relation to the app content you have just listened to with the research team. Each item is rated from 0 (strongly disagree) to 100 (strongly agree).

1. **The information I was given was understandable**

Strongly Disagree **Strongly Agree**

0 10 20 30 40 50 60 70 80 90 100

How could we improve?

2. **I feel that most 16–25-year-olds experiencing worry and/or anxiety would be able to work through this content**

Strongly Disagree **Strongly Agree**

0 10 20 30 40 50 60 70 80 90 100

How could we improve?

3. **There were concepts explained to me that I did not understand**

Strongly Disagree **Strongly Agree**

0 10 20 30 40 50 60 70 80 90 100

Can you remember which concepts you did not understand?

4. **Was there anything that you did which helped you create your image or make your image clearer?**

Please tell us more!

5. How did you find the stop worry practice task?

Please tell us more!

6. How likely would you be to come back to complete the next session?

Not at all likely **Very likely**

0 10 20 30 40 50 60 70 80 90 100

Why is this the case?

7. Were there any parts of the session which you did not like?

What section was this?

8. Were there any parts of the content which you would have preferred to be presented as animations or video?

Can you describe the content?

9. What were three things you learnt from this session?

10. Is there any other feedback you would like to give?

Please tell us more!

SESSION 7

Name:

Please rate each of the following items in relation to the app content you have just listened to with the research team. Each item is rated from 0 (strongly disagree) to 100 (strongly agree).

11. The information I was given was understandable

Strongly Disagree **Strongly Agree**

0 10 20 30 40 50 60 70 80 90 100

How could we improve?

12. I feel that most 16–25-year-olds experiencing worry and/or anxiety would be able to work through this content

Strongly Disagree **Strongly Agree**

0 10 20 30 40 50 60 70 80 90 100

How could we improve?

13. There were concepts explained to me that I did not understand

Strongly Disagree **Strongly Agree**

0 10 20 30 40 50 60 70 80 90 100

Can you remember which concepts you did not understand?

14. Was there anything that you did which helped you create your image or make your image clearer?

Please tell us more!

15. How did you find the stop worry practice task?

Please tell us more!

16. How likely would you be to come back to complete the next session?

Not at all likely **Very likely**

0 10 20 30 40 50 60 70 80 90 100

Why is this the case?

17. Were there any parts of the session which you did not like?

What section was this?

18. Were there any parts of the content which you would have preferred to be presented as animations or video?

Can you describe the content?

19. What were three things you learnt from this session?

20. Is there any other feedback you would like to give?

Please tell us more!

SESSION 8

Name:

Please rate each of the following items in relation to the app content you have just listened to with the research team. Each item is rated from 0 (strongly disagree) to 100 (strongly agree).

21. The information I was given was understandable

Strongly Disagree **Strongly Agree**

0 10 20 30 40 50 60 70 80 90 100

How could we improve?

22. I feel that most 16–25-year-olds experiencing worry and/or anxiety would be able to work through this content

Strongly Disagree **Strongly Agree**

0 10 20 30 40 50 60 70 80 90 100

How could we improve?

23. There were concepts explained to me that I did not understand

Strongly Disagree **Strongly Agree**

0 10 20 30 40 50 60 70 80 90 100

Can you remember which concepts you did not understand?

24. How did you find the stop worry practice in this session?

Please tell us more!

25. How did you find the added step of "getting back to life"?

Please tell us more!

26. How likely would you be to come back to complete the next session?

Not at all likely **Very likely**

0 10 20 30 40 50 60 70 80 90 100

Why is this the case?

27. Were there any parts of the session which you did not like?

What section was this?

28. Were there any parts of the content which you would have preferred to be presented as animations or video?

Can you describe the content?

29. What were three things you learnt from this session?

30. Is there any other feedback you would like to give?

Please tell us more!
